# Supplementary material for: Extraction, Isolation and Characterization of Mycosporine-like Amino Acids from Four Species of Red Macroalgae
Source: Mar Drugs. 2021 Oct 28;19(11):615. doi: 10.3390/md19110615 (PMC8625514; doi:10.3390/md19110615)
Supplement: Supplementary file 1 [file marinedrugs-19-00615-s001.zip › marinedrugs-1397920-supplementary.pdf]

Figure S1: HPLC spectrum

The following is HPLC spectra of the fraction T_1 which isolated from MAAs extracts from *Bangia fusco-purpurea*

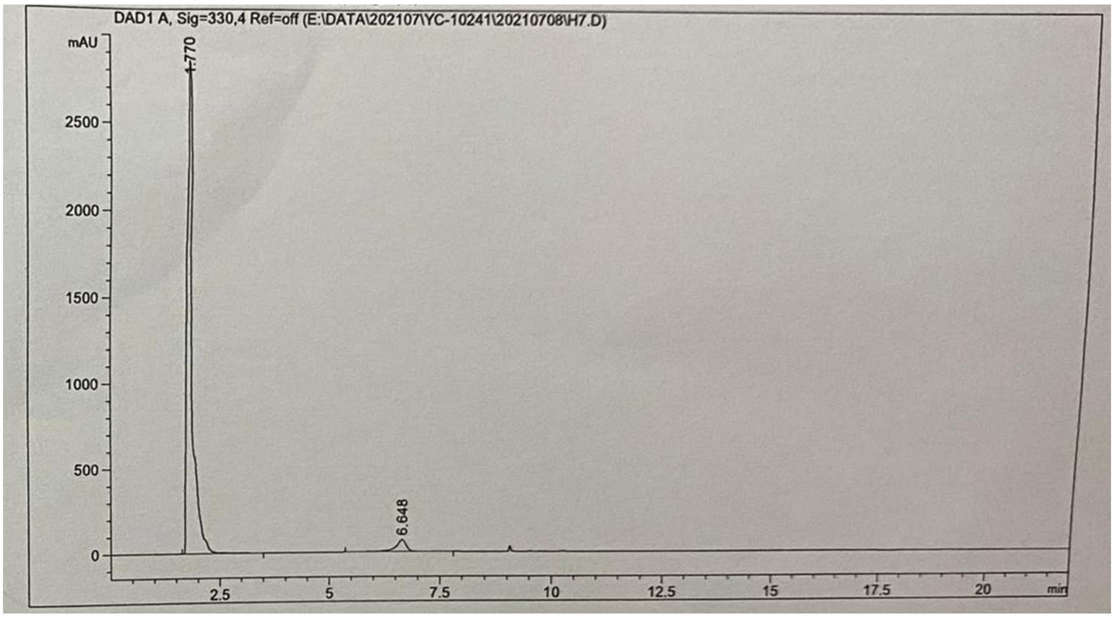

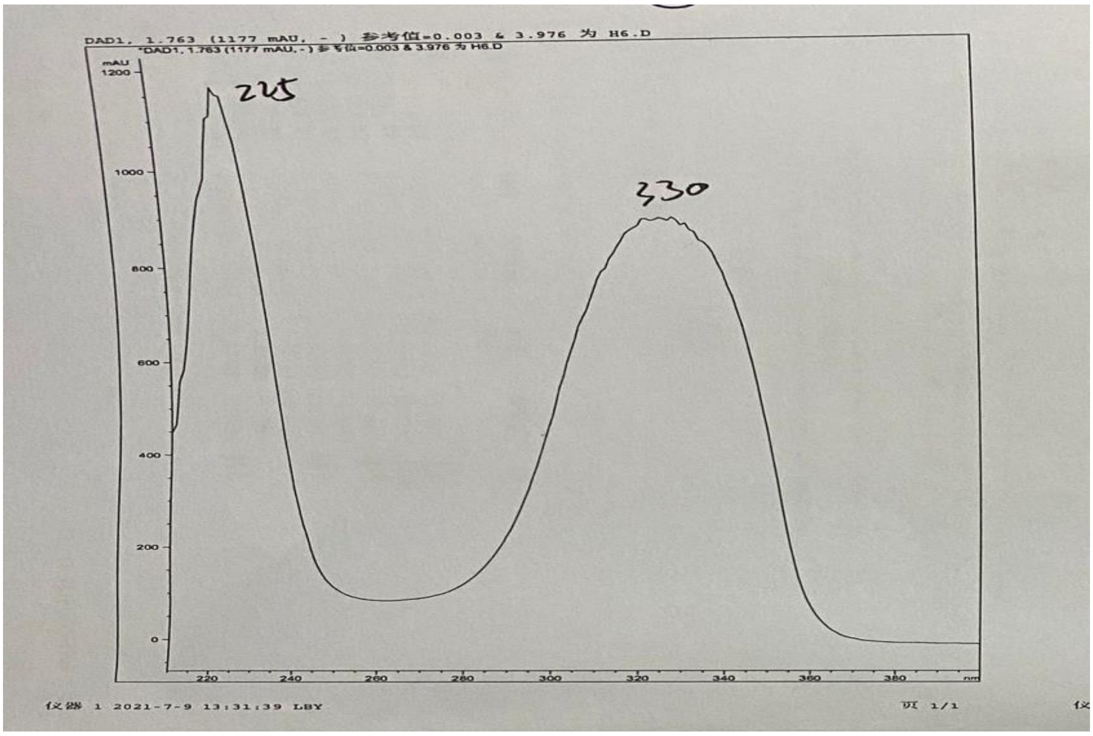

The following is HPLC spectra of the fraction J_1 which isolated from MAAs extracts from *Gracilaria* sp.

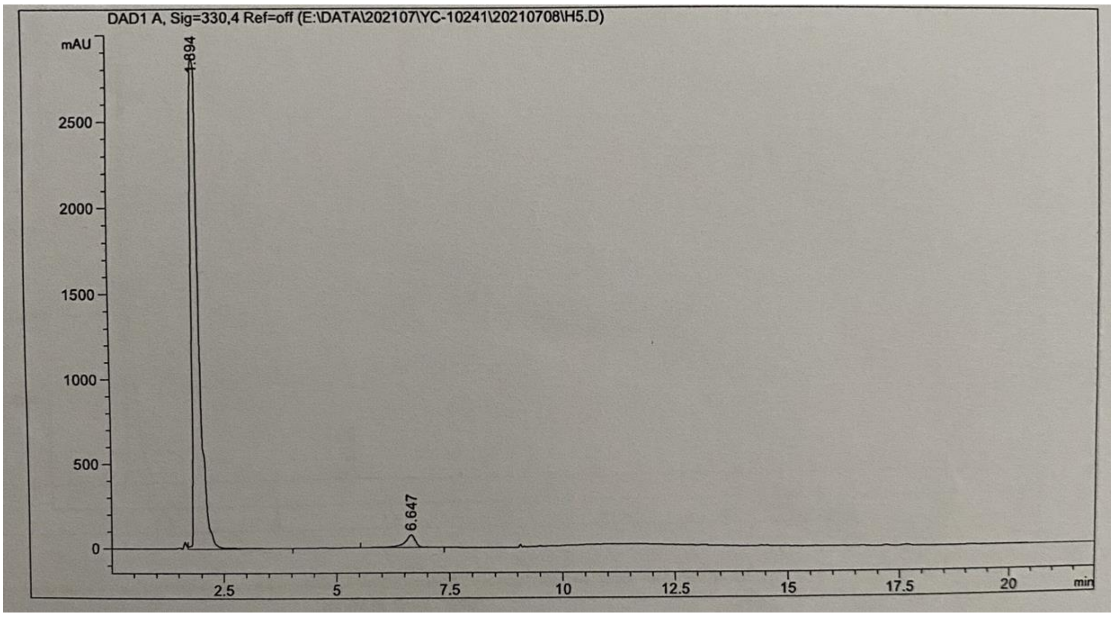

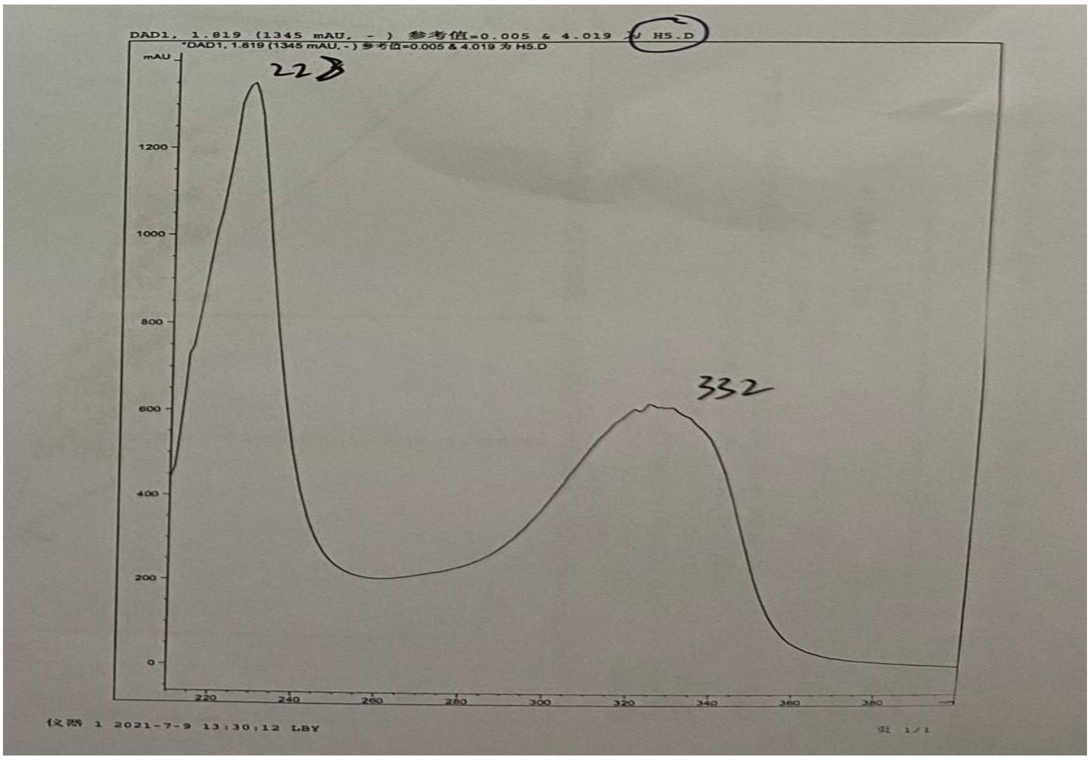

The following is HPLC spectra of the fraction J_2 which isolated from MAAs extracts from *Gracilaria* sp.

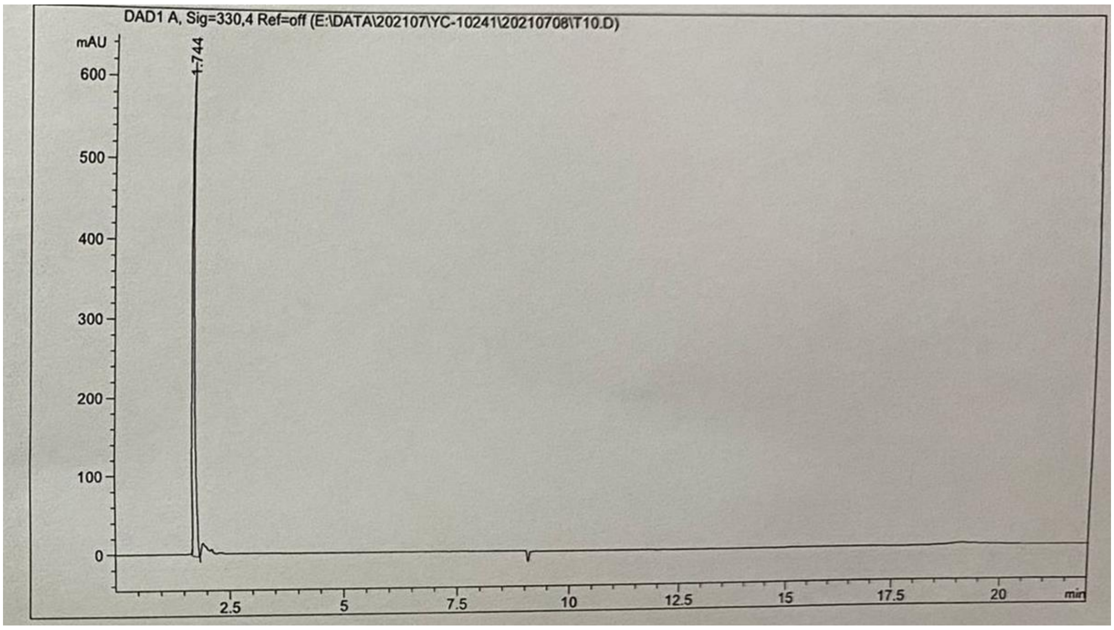

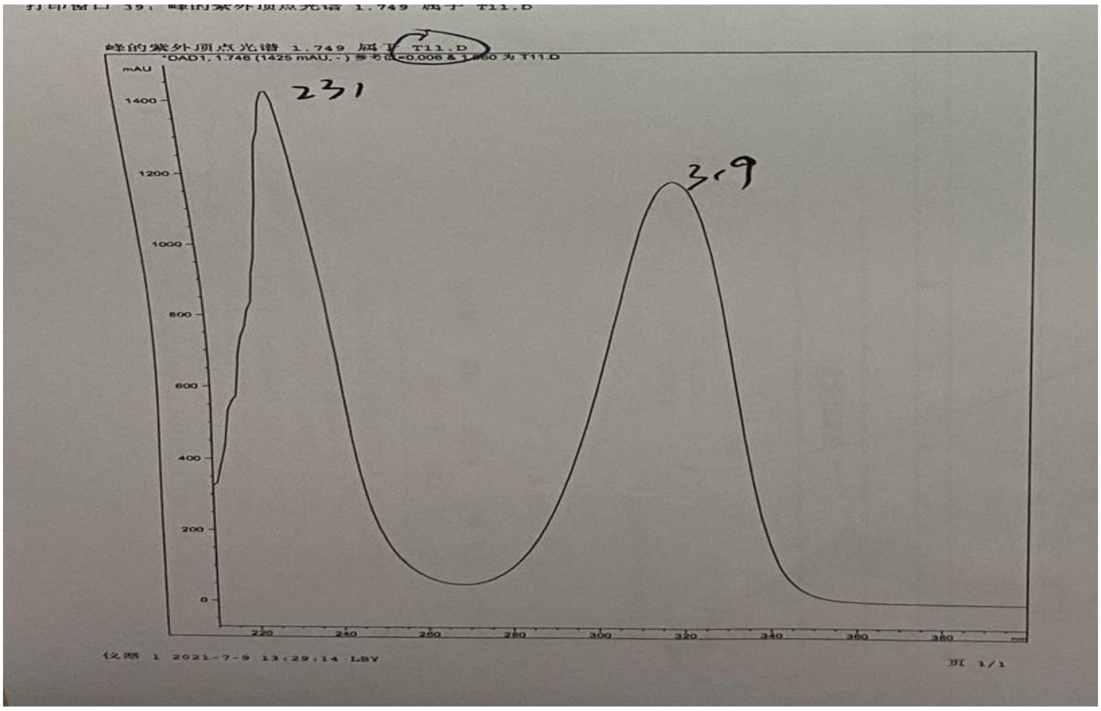

The following is HPLC spectra of MAAs extracts from *Gelidium amansii*

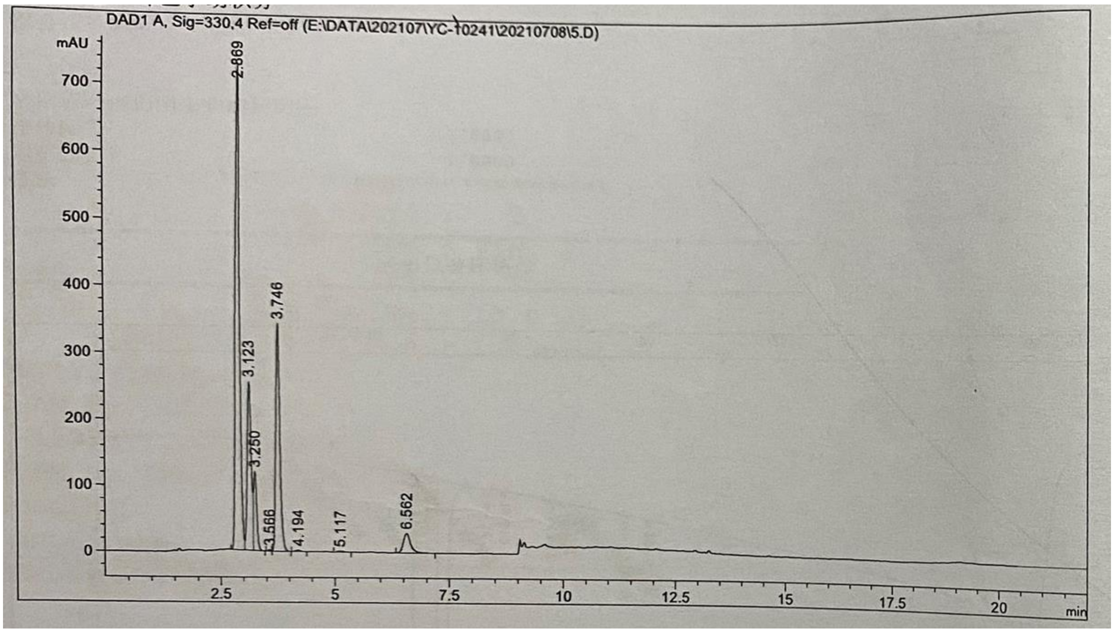

The following is HPLC spectra of MAAs extracts from *Gracilaria confervoides*

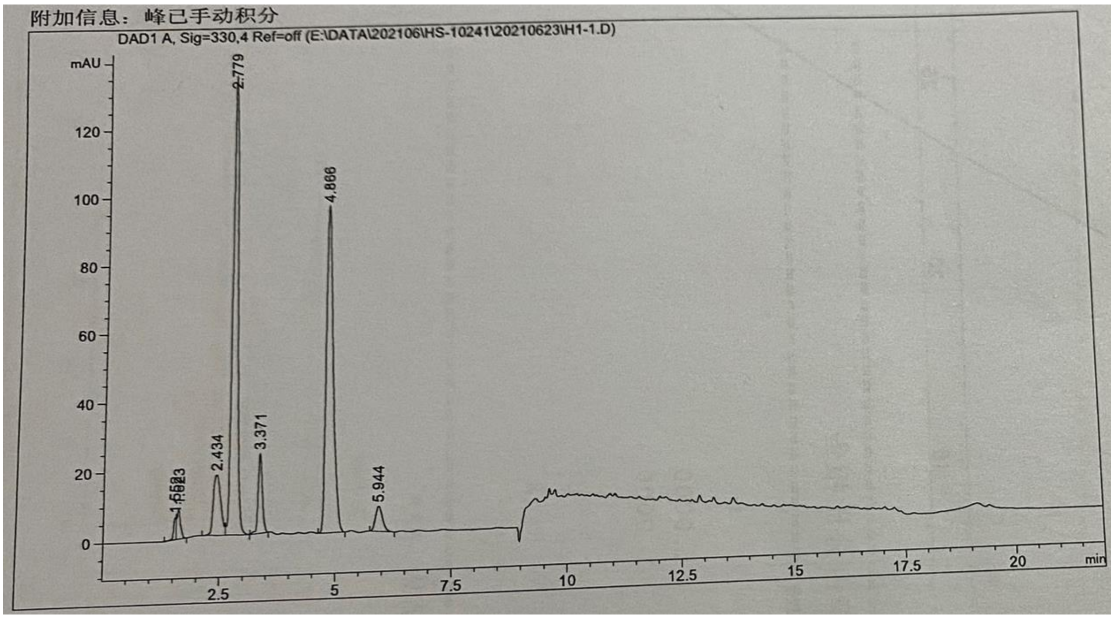

Figure S2: ESI-MS spectrum

The following is tandem mass spectra of one fraction (T_1) which isolated from MAAs extracts from *Bangia fusco-purpurea*

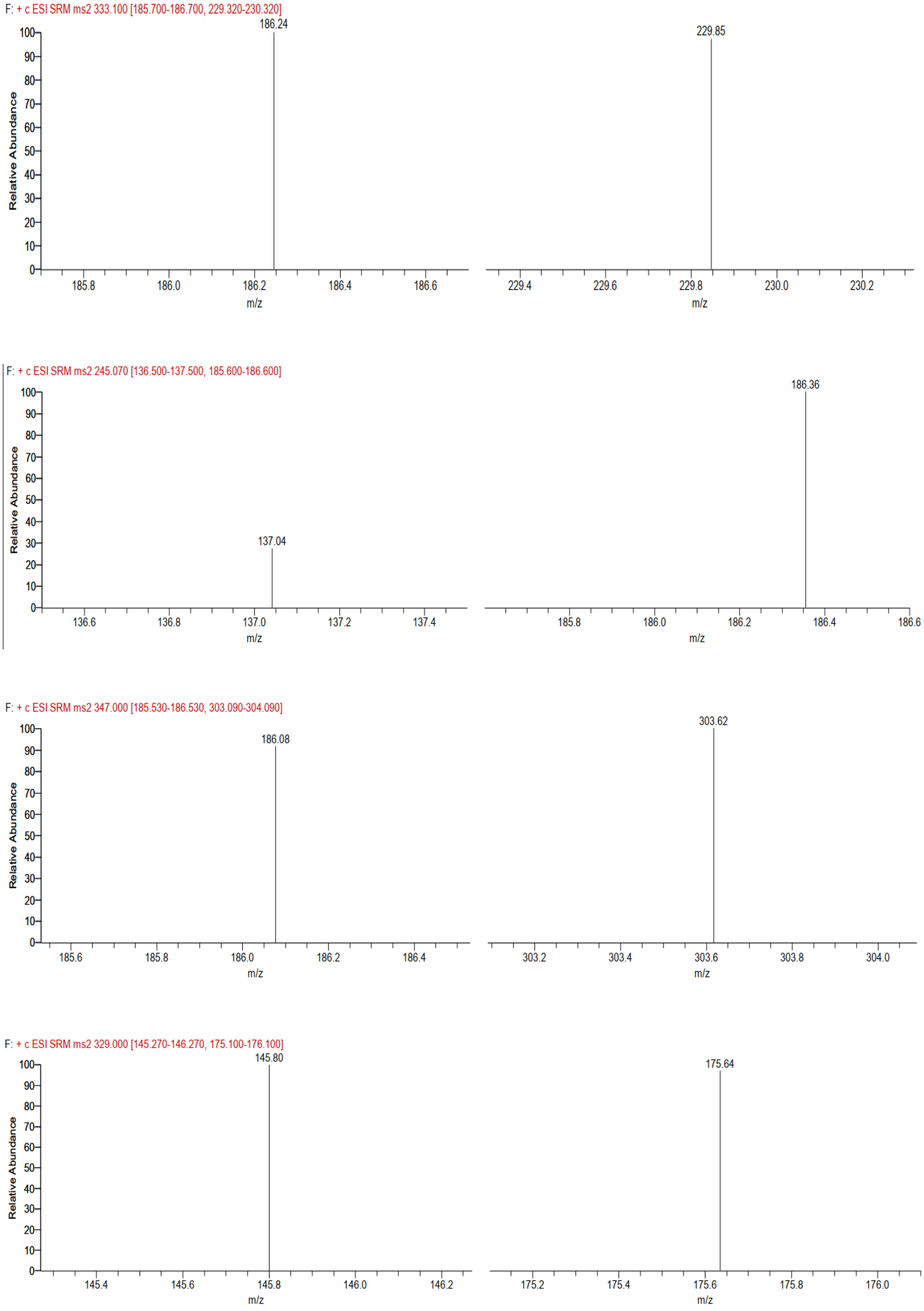

The following is the mass spectrum of two fractions (J_1 and J_2) which isolated from MAAs extracts from *Gracilaria* sp.

J_1

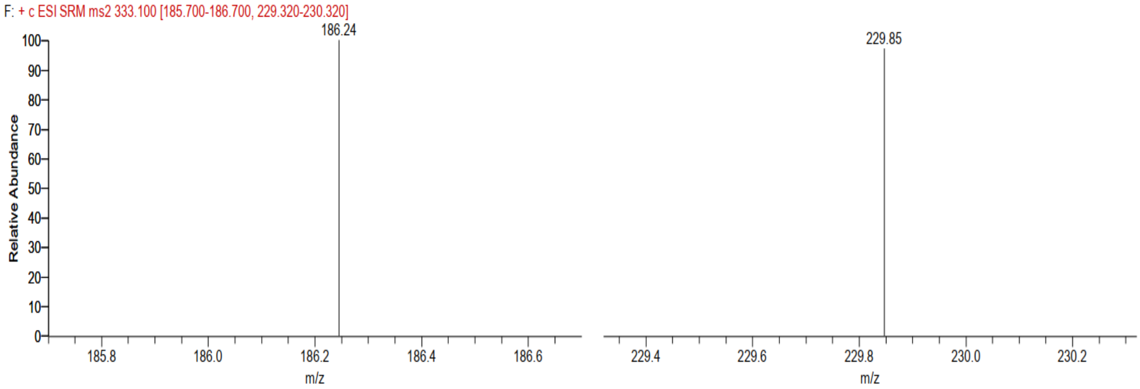

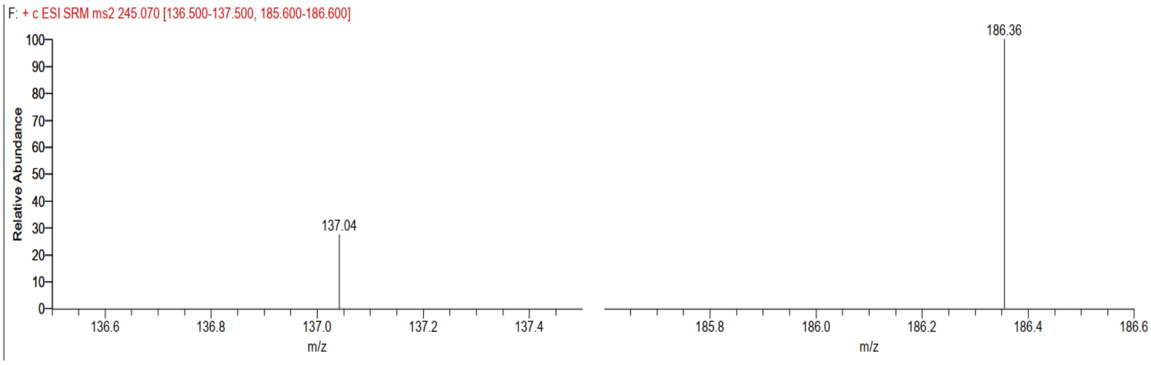

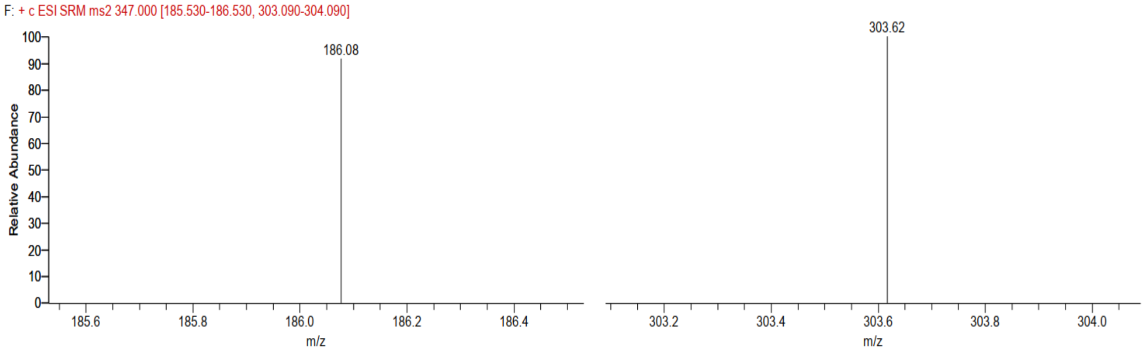

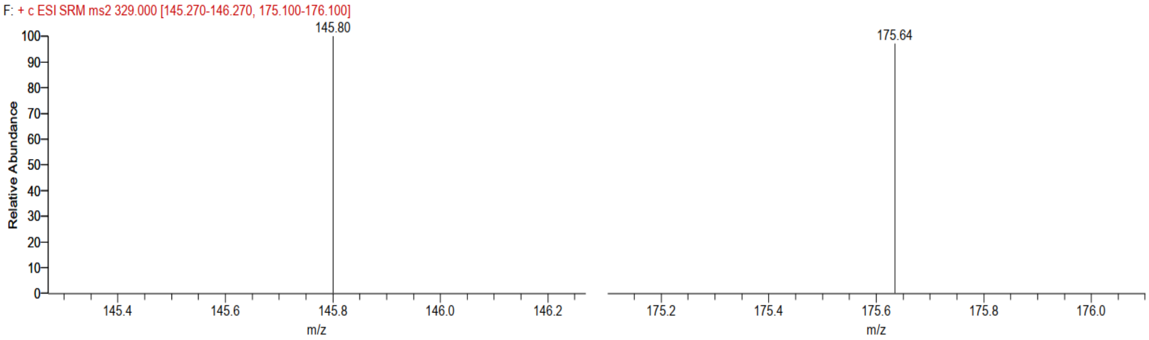

J_2

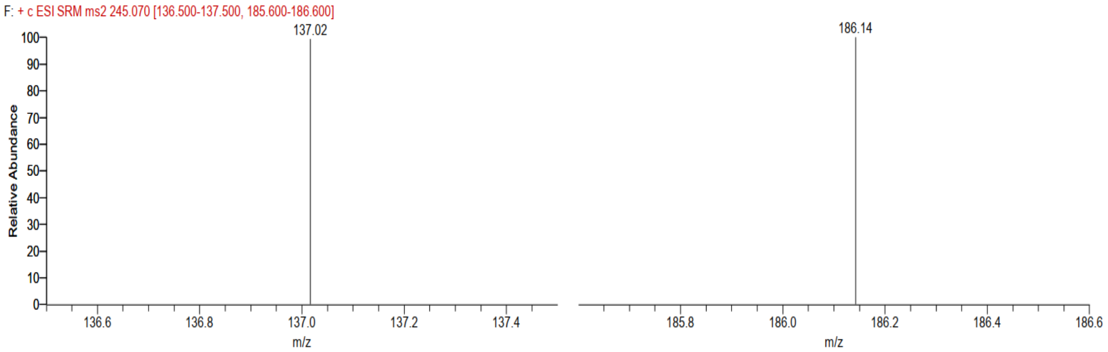
F: + c ESI SRM ms2 245.070 [136.500-137.500, 185.600-186.600]
137.02
186.14
Relative Abundance
100
90
80
70
60
50
40
30
20
10
0
136.6
136.8
137.0
137.2
137.4
185.8
186.0
186.2
186.4
186.6
m/z
m/z
